# A bibliometric analysis based on hotspots and frontier trends of positron emission tomography/computed tomography utility in bone and soft tissue sarcoma

**DOI:** 10.3389/fonc.2024.1344643

**Published:** 2024-06-21

**Authors:** Feifan Xiang, Yue Zhang, Xiaoqi Tan, Jintao Zhang, Tengfei Li, Yuanzhuo Yan, Wenzhe Ma, Yue Chen

**Affiliations:** ^1^ The State Key Laboratory of Quality Research in Chinese Medicine, Macau University of Science and Technology, Macao, Macao SAR, China; ^2^ Department of Orthopedic, Affiliated Hospital of Southwest Medical University, Luzhou, China; ^3^ Department of Nuclear Medicine, Affiliated Hospital of Southwest Medical University, Luzhou, China; ^4^ Department of Dermatology, Affiliated Hospital of Southwest Medical University, Luzhou, China; ^5^ Nuclear Medicine and Molecular Imaging Key Laboratory of Sichuan Province, Luzhou, China; ^6^ Institute of Nuclear Medicine, Southwest Medical University, Luzhou, China

**Keywords:** PET/CT, bone and soft tissue sarcoma, ^18^F-FDG, bibliometrics, hotspots, development trends

## Abstract

**Purpose:**

This study aimed to analyze articles on the diagnosis and treatment of bone and soft tissue sarcoma using positron emission tomography (PET)/computed tomography (CT) published in the last 13 years. The objective was to conduct a bibliometric analysis and identify the research hotspots and emerging trends.

**Methods:**

Web of Science was used to search for articles on PET/CT diagnosis and treatment of bone and soft tissue sarcoma published from January 2010 to June 2023. CiteSpace was utilized to import data for bibliometric analysis.

**Results:**

In total, 425 relevant publications were identified. Publications have maintained a relatively stable growth rate for the past 13 years. The USA has the highest number of published articles (139) and the highest centrality (0.35). The UDICE-French Research Universities group is the most influential institution. BYUN BH is a prominent contributor to this field. The Journal of Clinical Oncology has the highest impact factor in the field.

**Conclusion:**

The clinical application of PET/CT is currently a research hotspot. Upcoming areas of study concentrate on the merging of PET/CT with advanced machine learning and/or alternative imaging methods, novel imaging substances, and the fusion of diagnosis and therapy. The use of PET/CT has progressively become a crucial element in the identification and management of sarcomas. To confirm its efficacy, there is a need for extensive, multicenter, prospective studies.

## 1 Introduction

Bone and soft tissue sarcomas (STS) are a group of heterogeneous malignancies originating from mesenchymal tissues (1, 2). STS is rare, comprising approximately 1% of adult solid tumors. Nevertheless, osteosarcoma (OS) remains the predominant initial malignant bone neoplasm in children and teenagers, constituting around half of all pediatric tumors, with Ewing’s sarcoma (ES) following closely (3–5). Sarcoma, as a malignant tumor, typically presents with a 5-year survival rate of around 65% and is commonly detected in later stages (6–9). Therefore, early detection and accurate assessment of the scope of the disease to take targeted treatment measures for patients at different stages are important for patient prognosis (10, 11).

Managing and treating sarcoma requires a multidisciplinary approach and the utilization of different imaging techniques. In response to new personalized treatment protocols, imaging methods are quickly advancing and are crucial in diagnosing, staging, monitoring treatment response, and monitoring relapse (3). Positron emission tomography (PET), a functional imaging method, utilizes positron emission isotopes labeled on specific molecules to visualize tissues or processes of interest. The combination of PET and computed tomography (CT) enables the identification of the exact location of radioactive tracer buildup and the simultaneous detection of anatomical and structural abnormalities (12).

To assess sarcomas, ^18^F-FDG, also known as 2-deoxy-2-(^18^F) fluoro-D-glucose, is extensively utilized as a metabolic tracer. Nevertheless, PET/CT imaging is not restricted to ^18^F-FDG alone, as there have been investigations into alternative tracers such as ^18^F-fluoronidazole (FMISO).This may provide additional diagnostic and prognostic indicators for hypoxia and the proliferation of sarcomas (9, 13, 14). As a result, PET/CT is being increasingly used in various clinical conditions and has a significant impact on the assessment of sarcomas, including guiding biopsies, staging diseases, and evaluating responses (9). Furthermore, the integration of PET and magnetic resonance imaging (PET/MRI) has been extensively researched and holds significant potential owing to its minimal radiation exposure and exceptional differentiation of soft tissues (15, 16). Considering these benefits, it is important to summarize the functional and research hotspots of PET imaging applied to sarcoma in the existing scientific literature. This consolidation aims to enhance the efficacy of sarcoma treatment and other clinical conditions, while fostering the advancement of imaging techniques.

In the age of big data, bibliometrics represents a comprehensive knowledge system capable of analyzing knowledge carriers in specific domains (17) in an objective and quantitative manner. It aids in summarizing the research hotspots and development trends in the areas of bone sarcoma and STS. CiteSpace, a Java program, utilizes bibliometrics to visually display the arrangement and dissemination of knowledge within a particular domain through a graphical representation (18). At present, there is no visual analysis research on PET/CT application in the bone sarcoma and STS fields at home or abroad. Hence, utilizing the pertinent publications available in the primary repository of the Web of Science (WOS), this investigation employs the CiteSpace application to illustrate and analyze the focal points of research in this domain through visual diagrams from diverse perspectives, aiming to anticipate future research directions. The findings of this research offer a valuable guide for enhancing the management of sarcomas, broadening the utilization of PET/CT, and fostering the advancement of novel imaging techniques.

## 2 Materials and methods

### 2.1 Data collection

The WOS core database offers a carefully curated selection of academic journals and publications, enabling access to comprehensive details and references for every article. To ensure data integrity and accuracy, we used the MeSH Browser (https://meshb.nlm.nih.gov/) to determine the search keywords. We downloaded all the data for analysis from the core database of WOS. The retrieval strategy included (TS=(OS) OR TS=(Osteogenic Sarcoma) OR TS=(Osteosarcoma Tumor) OR TS=(soft tissue sarcoma)) AND (TS=(PET/CT) OR TS=(Positron Emission Tomography/Computed Tomography)), with the retrieval period set from January 1, 2010 to June 9, 2023.Only English literature was retrieved. A total of 558 search results were saved in plain-text format and imported to CiteSpace for visualization. All data were extracted on 2023–06-09.

### 2.2 Bibliometric analysis

**Figure 1:**
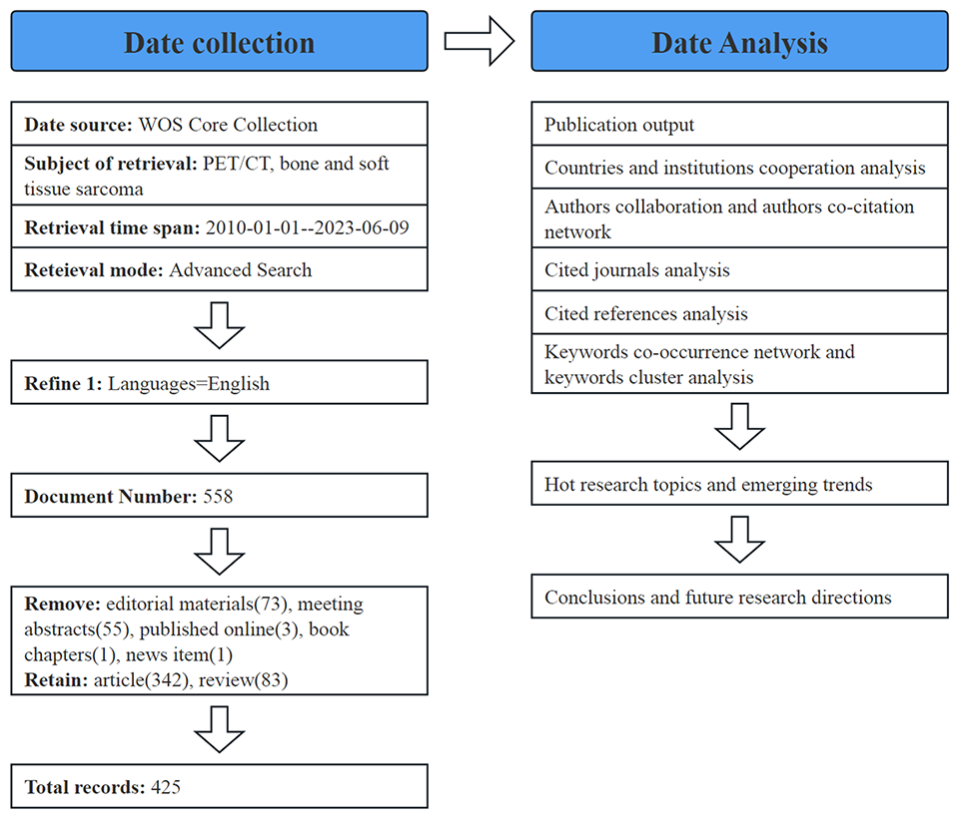
Stages of bibliometric analysis of PET/CT utility in bone and soft tissue sarcoma.

All the literature data retrieved were imported into CiteSpace; only two types of literature, “Article” and “Review,” were retained, and literature such as letters, editorial materials, conference abstracts, and online publications were deleted. In total, 425 valid studies were obtained, including 342 articles and 83 reviews (**Figure 1**). Subsequently, this information was utilized to create visual knowledge diagrams encompassing nations, publications, keyword co-occurrence, and clustering. In the graph, different nodes represent different research objects, and the range of nodes represents the number of publications or the frequency of citations. Cooperation or co-citation relationships are represented by wiring, with different years indicated by various colors within the nodes. A node with a high degree of centrality is denoted by a purple outer edge.

## 3 Results

### 3.1 Annual publication volume analysis

**Figure 2:**
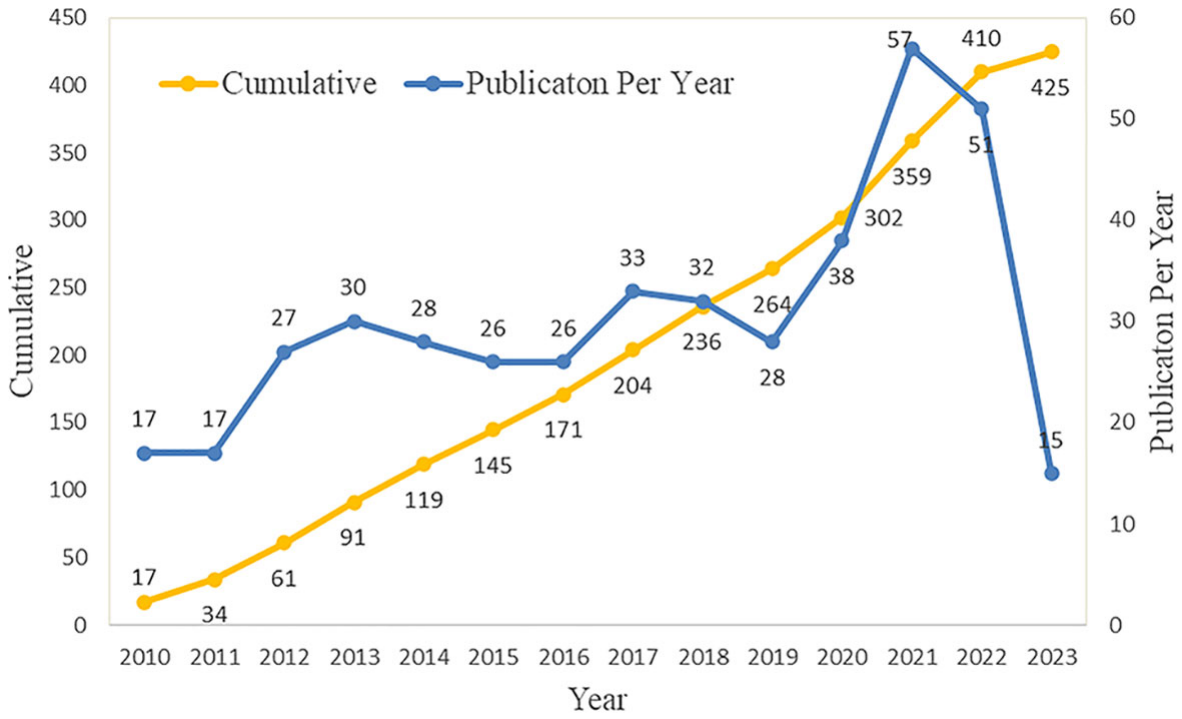
The annual output of PET/CT utility in bone and soft tissue sarcoma.

The annual number of published documents and trend of published documents can objectively and quantitatively reflect the developmental status of specific fields at different stages (19). Although there have been variations in the last 13 years, the rate of expansion in publications has maintained a relatively consistent level. The rapid advancement of PET/CT is closely interconnected with this. PET/CT now has a broader scope in the detection and management of tumors, extending beyond the identification of benign and malignant tumors. This advanced technology is crucial in diagnosing, classifying, predicting outcomes, and assessing treatment effectiveness for sarcomas (20). Furthermore, with the popularity of PET/CT, more patients benefit from it, which has become a driving force in promoting the continuous improvement of PET/CT functions and the development of new imaging technologies. The number of articles published in 2023 has decreased, with only 15 articles; however, it should be noted that the current study only included literature published in the first half of 2023.Further investigation is warranted to explore the potential of PET/CT as a promising imaging technique for the diagnosis and treatment of sarcoma. Additional large-scale prospective experiments are needed. It is believed that it will still be a research hotspot, and more research results will be produced in the future (**Figure 2**).

### 3.2 Contribution analysis of countries and institutions

**Figure 3:**
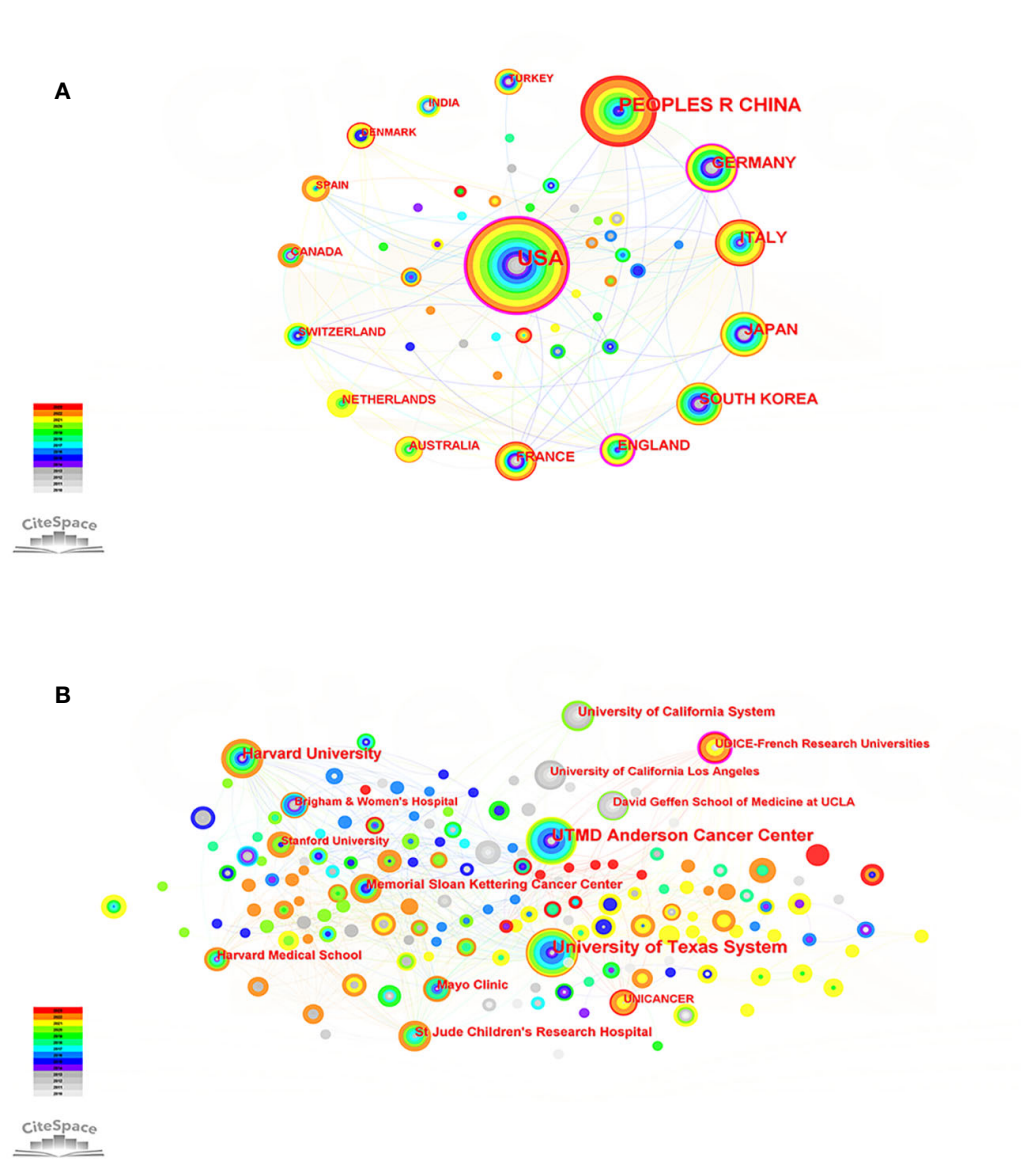
**(A)** Country map of the PET/CT utility in bone and soft tissue sarcoma from 2010 to 2023. **(B)** Network map of institutional cooperation of the PET/CT utility in bone and soft tissue sarcoma from 2010 to 2023.

We mapped the co-occurrence of national publications in the field of PET/CT application to sarcoma. A country is symbolized by a node, and the more extensive the node range, the higher the quantity of published articles. Over the past 13 years, 159 countries have contributed to this field. The United States (139 articles, 32.71%) emerged as the primary contributor, closely followed by China (73 articles, 17.18%) and Germany (37 articles, 8.71%). The USA holds the highest number of citations (2596), making it a significant indicator of the country’s influence in a particular scientific domain. Germany (33.92) had the highest average citation rate (AC), indicating that it publishes many high-quality studies. Furthermore, the violet perimeter of the nodes in the diagram signifies that the nation possesses a greater level of centrality; the greater the centrality, the stronger the linkage with other nations. The United States (0.35) maintains its position as the most central country. In general, despite having a higher number of articles, Asian nations like China and Japan exhibit slightly lower influence in terms of articles and collaboration with other countries (**Figure 3A**; **Supplementary Table 1**).

The top three institutions, based on the number of publications, were the University of Texas System (30 articles), UTMD Anderson Cancer Center (28 articles), and Harvard University (18 articles). The UDICE-French Research Universities (0.13) consortium holds the utmost centrality, investigating the significance of ^18^F-FDG PET/CT in the identification, classification, and prediction of rhabdomyosarcoma, and has gained extensive acknowledgement (21–23). It should be noted that, apart from this establishment, the remaining top 10 highly productive establishments originate from the United States. The United States has played a crucial role in promoting the utilization of PET/CT in the sarcoma field and has served as a conduit for fostering extensive collaboration with institutions in different nations (**Figure 3B**; **Supplementary Table 2**).

### 3.3 Contribution analysis of authors and co-cited authors

In this area of study, more than 407 authors have published relevant articles in the past 13 years, of which 34 have published at least three articles. The top two—Kong, Chang-Bae (seven articles) and Lim, Ilhan (seven articles)—collaborated multiple times with Byun, Byung Hyun (six articles), who ranked fourth, focusing on using ^18^F-FDG PET/CT or combining other imaging techniques, to monitor the therapeutic response of OS neoadjuvant chemotherapy. It has been proposed that the histological response of OS neoadjuvant chemotherapy can be precisely anticipated by FDG PET following a single treatment session, with metabolic tumor volume (MTV) and total lesion glycolysis (TLG) serving as autonomous indicators of histological response (24, 25). Anderson, Peter M et al. (26) used PET/CT was utilized to assess the effectiveness of Robatumumab in treating OS and ES, demonstrating that insulin-like growth factor receptor-1 remains a compelling target for therapy. Notably, Jiang Huiyan, the sole writer affiliated with Northeastern University in China, contributed to the release of multiple papers in 2023 that focused on the integration of PET/CT and deep learning. These articles aimed to enhance the precision of tumor segmentation objectives and advance the progress of PET/CT imaging (27–29) (**Table 1**; **Supplementary Figure 1A**).

**Table 1:** Top 10 most productive authors.

Rank	Author	NP	Institution	Country
1	Kong, Chang-Bae	7	Korea Institute of Radiological & Medical Sciences	SOUTH KOREA
2	Lim, Ilhan	7	Korea Institute of Radiological & Medical Sciences	SOUTH KOREA
3	Daw, Najat C	7	UTMD Anderson Cancer Center	USA
4	Byun, Byung Hyun	6	National Cancer Center - Korea	SOUTH KOREA
5	Jeon, Dae-Geun	5	National Cancer Center - Korea	SOUTH KOREA
6	Lim, Sang Moo	5	National Cancer Center - Korea	SOUTH KOREA
7	Cho, Wan Hyeong	5	National Cancer Center - Korea	SOUTH KOREA
8	Anderson, Peter M	5	Cleveland Clinic Foundation	USA
9	Amini, Behrang	5	UTMD Anderson Cancer Center	USA
10	Jiang, Huiyan	5	Northeastern University	China

NP, total number of publications.

Co-cited authors are those who are cited together in one or more papers simultaneously. Experiments conducted by BENZ MR (frequency: 77, 0.13) demonstrated that FDG PET/CT scans have the ability to anticipate the histopathological response to neoadjuvant chemotherapy in high-level STS and can offer guidance for treatment choices in individuals with sarcoma. Therefore, the author’s research has the highest centrality and this is also the author with the second-highest citation frequency (30). TATEISHI U (frequency: 84), the author with the highest number of citations, employed PET/CT to observe the structural and metabolic alterations of bone metastases in individuals diagnosed with metastatic breast cancer following systemic therapy. The author discovered that the decrease in standardized uptake value (SUV) served as a standalone indicator for treatment response, establishing the groundwork for subsequent investigations (31). In pediatric oncology, the ^18^F-FDG PET/CT imaging guide was authored by the fourth author, FRANZIUS C (frequency: 69). This guide includes details about indications, acquisition, processing, and interpretation, and offers a structure for nuclear medicine teams to follow in their daily practice (32) (**Table 2**; **Supplementary Figure 1B**).

**Table 2:** Top 10 cited authors and their highly-cited articles.

Rank	Cited author	Frequency	Highly-Cited Reference	Cited author	Centrality
1	TATEISHI U	84	Bone metastases in patients with metastatic breast cancer: Morphologic and metabolic monitoring of response to systemic therapy with integrated PET/CT	BENZ MR	0.13
2	BENZ MR	77	FDG-PET/CT Imaging Predicts Histopathologic Treatment Responses after the Initial Cycle of Neoadjuvant Chemotherapy in High-Grade Soft-Tissue Sarcomas	HAWKINS DS	0.11
3	BYUN BH	70	Combination of F-^18^-FDG PET/CT and Diffusion-Weighted MR Imaging as a Predictor of Histologic Response to Neoadjuvant Chemotherapy: Preliminary Results in Osteosarcoma	BIELACK SS	0.11
4	FRANZIUS C	69	Guidelines for F-^18^-FDG PET and PET-CT imaging in paediatric oncology	COSTELLOE CM	0.1
5	HAWKINS DS	64	Ewing Sarcoma: Current Management and Future Approaches Through Collaboration	BYUN BH	0.09
6	COSTELLOE CM	63	F-^18^-FDG PET/CT as an Indicator of Progression-Free and Overall Survival in Osteosarcoma	FRANZIUS C	0.09
7	VOLKER T	57	Positron emission tomography for staging of pediatric sarcoma patients: Results of a prospective Multicenter trial	VOLKER T	0.09
8	EARY JF	56	Spatial Heterogeneity in Sarcoma F-^18^-FDG Uptake as a Predictor of Patient Outcome	EVILEVITCH V	0.09
9	BRENNER W	44	Monitoring Primary Systemic Therapy of Large and Locally Advanced Breast Cancer by Using Sequential Positron Emission Tomography Imaging With [F-^18^] Fluorodeoxyglucose	WAHL RL	0.09
10	IM HJ	43	Prognostic value of volumetric parameters of F-^18^-FDG PET in non-small-cell lung cancer: a meta-analysis	TATEISHI U	0.08

### 3.4 Analysis of cited journals

**Figure 4:**
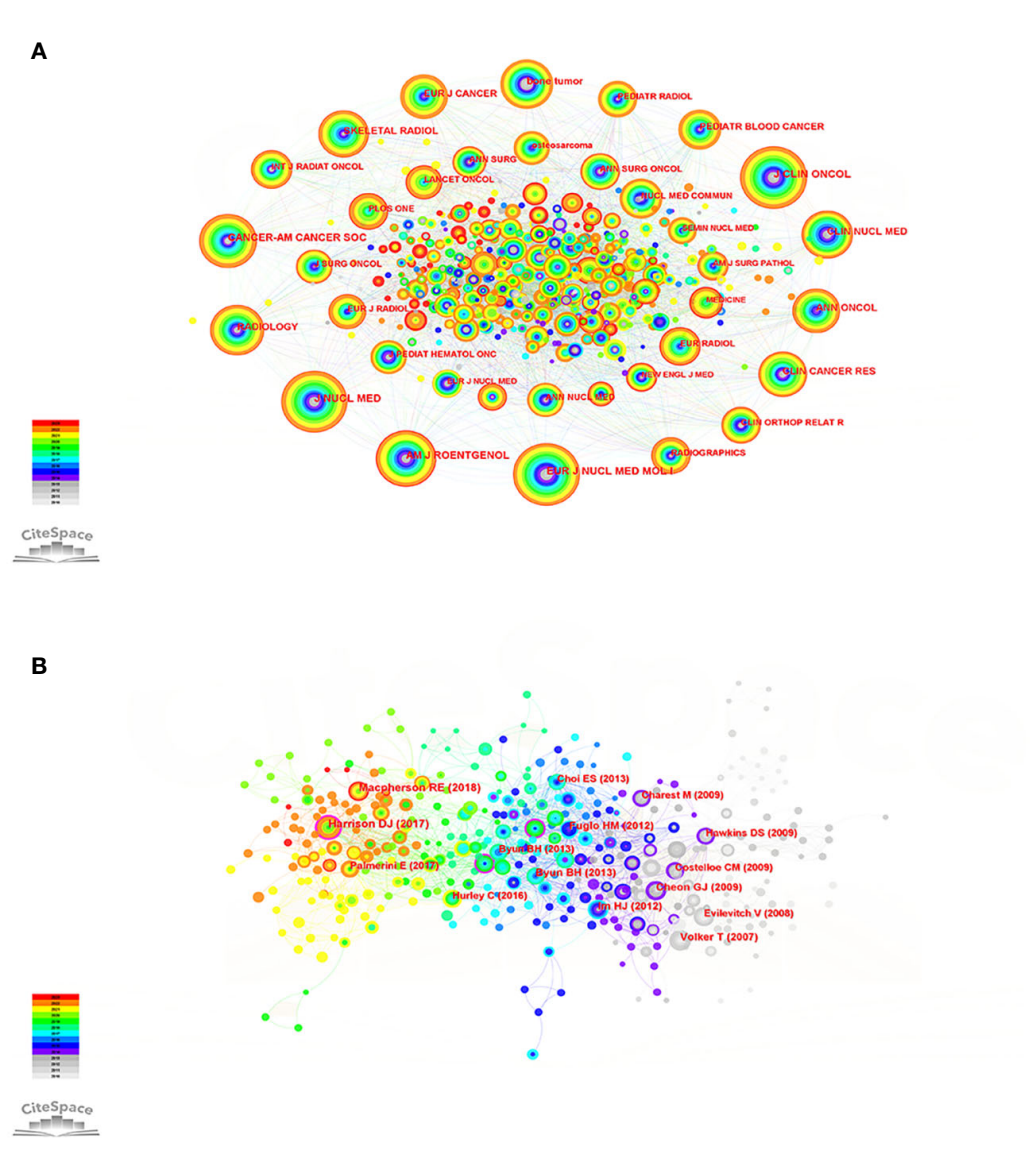
**(A)** Co-citation network of cited journals of the PET/CT utility in bone and soft tissue sarcoma from 2010 to 2023. **(B)** Co-citation network of cited references of the PET/CT utility in bone and soft tissue sarcoma from 2010 to 2023.

To comprehend the dissemination of the knowledge repository regarding PET/CT applications for sarcoma, we conducted a visual analysis of the co-occurrence network in highly referenced journals (**Figure 4A**). Evaluating the quality and influence of a journal is crucial, and two significant criteria for this are the journal’s impact factor (IF) and journal citation reports (JCR) zoning. The journal with the highest frequency of citations is the《JOURNAL OF NUCLEAR MEDICINE》(frequency: 257), and numerous articles published in this journal demonstrate the varied application value of PET/CT in the sarcoma field from various perspectives. This serves as evidence that PET/CT is a highly promising imaging technology (33–35). The《JOURNAL OF CLINICAL ONCOLOGY》(frequency: 254, 45.3) holds the highest impact factor (IF) and has published numerous high-quality and recent protocols and findings regarding bone sarcoma and STS treatment (36, 37). According to the《EUROPEAN JOURNAL OF NUCLEAR MEDICINE AND MOLECULAR IMAGING》(frequency: 245), ^68^Ga-DOTA-FAPI-04 PET/CT outperforms ^18^F-FDG in detecting recurrent STS lesions, including liposarcoma. This innovative imaging technique holds promise for monitoring STS recurrence. In general, in the field of bone sarcoma and STS, PET/CT imaging has more application value than ordinary imaging methods. Further, the imaging technology is still improving, and there are more possibilities for us to explore (**Supplementary Table 3**).

### 3.5 Analysis of cited references

Through the examination of the crucial points in the depicted network of referenced literature, we can uncover the shift in research focus within this particular field and identify the pivotal literature that contributes significantly to this transformation. The top 10 cited studies have promoted the development of PET/CT application in the sarcoma field and influenced the change of research direction. A comprehensive retrospective study demonstrated that PET/CT can provide substantial advantages in the standard CT/MRI evaluation and re-evaluation of advanced bone sarcoma and STS (38). However, Harrison et al. (39) note that there are conflicting clinical trial results for its use in predicting outcomes in pediatric sarcoma staging. Additional extensive research is required to fully ascertain the optimal way to integrate it into forthcoming therapeutic protocols for pediatric sarcomas. The effectiveness of predicting the histological response to neoadjuvant chemotherapy may be improved by combining PET/CT with diffusion-weighted imaging (DWI), as suggested (40). Moreover, FDG PET/CT has the capability to detect individuals who are prone to developing resistance to chemotherapy based on the highest SUV (SUVmax) (41). To summarize, additional future and multicenter assessments of PET/CT are necessary to establish the true prognostic significance and cost-efficiency of PET/CT in guiding the medical treatment of clinically intricate and diverse high-grade sarcomas (**Figure 4B**; **Supplementary Table 4**).

### 3.6 Analysis of keywords

**Figure 5:**
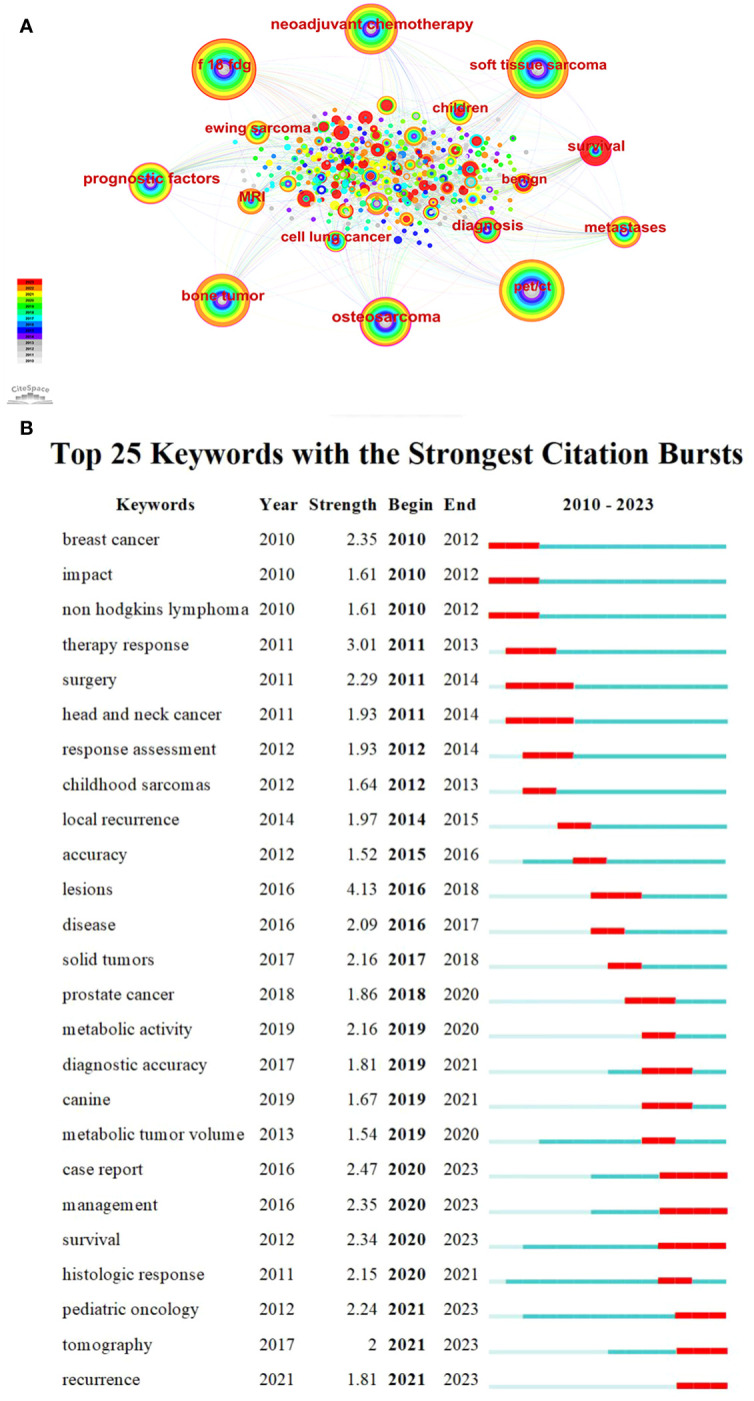
**(A)** Keywords co-occurrence graph of the PET/CT utility in bone and soft tissue sarcoma from 2010 to 2023. **(B)** Top 20 keywords with the strongest citation bursts.

By conducting a visual analysis of keywords (**Figure 5A**), it is possible to gain initial insights into the research focal points and emerging trends in this particular area. The red ring in the node represents the keyword had a citation burst (**Figure 5B**). The more powerful the burst, the more significant the impact of the keyword, which is the current focus of research. The keywords that have more burst strength in recent years reflect the development trend of this field.

It can be seen that “^18^F-FDG” (frequency: 198) is the keyword with the second highest frequency after PET/CT (frequency: 203). Sarcoma is commonly evaluated using FDG, a radioactive substance similar to glucose and widely used as a metabolic tracer (42). The utilization of ^18^F-FDG PET/CT is applicable for the purposes of staging, identifying recurrence, tracking response to chemotherapy, and forecasting prognosis (43). The terms “soft tissue sarcoma” (frequency: 179), “bone tumor” (frequency: 141), “osteosarcoma” (frequency: 117), and “Ewing sarcoma” (frequency: 37) demonstrate extensive evidence of the broad indications of ^18^F-FDG PET/CT in both bone sarcoma and STS (3, 44, 45). Further, “neoadjuvant chemotherapy” (NCT, centrality: 0.19) has the second highest centrality, demonstrating the importance of NCT for the treatment of sarcoma, and the tumor response to NCT provides us with further information relevant to each patient’s biological behavior and helps to predict the ultimate oncology outcome (46, 47). Furthermore, the OS exhibits a strong inclination to metastasize and disseminate, with the lungs accounting for 80% of metastatic occurrences. Hence, it is crucial to precisely evaluate the extent of the illness, particularly metastatic conditions, and identify any signs of relapse at an early stage. The research focus has shifted toward non-small cell lung cancer (centrality: 0.13) and metastases (centrality: 0.11) in recent years (48–50).

It is important to mention that the term “case report” (strength: 2.47) has the highest burst strength among keywords in recent years, with a total of 77 case reports included in our analysis of literature. These case reports prove the application value of radioactive tracer and radionuclide therapy in the treatment of common diseases to rare diseases; while these are interesting and could lead to some new hypotheses, they do not contribute much to exploring the value of PET/CT imaging for truly specific, standard clinical applications (51, 52). This reminds us of the need for a shift in research types. “Management” (strength: 2.35) is the keyword with the second highest burst strength. Treatment for sarcomas is usually multimodal, including systemic chemotherapy and aggressive surgical resection (39). In recent times, as the idea of combined diagnosis and treatment became deeply rooted in the public consciousness, ^18^F-FDG PET/CT has emerged as a vital component in the management of sarcoma. It offers valuable anatomical and functional data, leading to alterations in treatment approaches and enhancing survival rates by enabling more precise staging and evaluation of response (53). Therefore, how to better use PET/CT to improve the survival rate of sarcoma has become a recent hotspot, and also a likely future development trend. “Survival” (strength: 2.34) obtained the third highest burst strength (**Figure 5**; **Table 3**).

**Table 3:** Top 15 keywords in terms of frequency and centrality.

Rank	Keywords	Frequency	Keywords	Centrality
1	PET/CT	203	osteosarcoma	0.22
2	^18^F-FDG	198	neoadjuvant chemotherapy	0.19
3	soft tissue sarcoma	179	diagnosis	0.16
4	bone tumor	141	prognostic factors	0.14
5	neoadjuvant chemotherapy	132	bone tumor	0.13
6	osteosarcoma	117	Non-small cell lung cancer	0.13
7	prognostic factors	88	gene expression	0.12
8	metastases	53	survival	0.11
9	survival	49	metastases	0.11
10	Ewing sarcoma	37	^18^F-FDG	0.09
11	diagnosis	36	follow up	0.09
12	MRI	36	MRI	0.08
13	children	36	breast cancer	0.08
14	gene expression	29	soft tissue sarcoma	0.07
15	Non-small cell lung cancer	28	Ewing sarcoma	0.07

### 3.7 Analysis of hotspots based on keyword clustering

**Figure 6:**
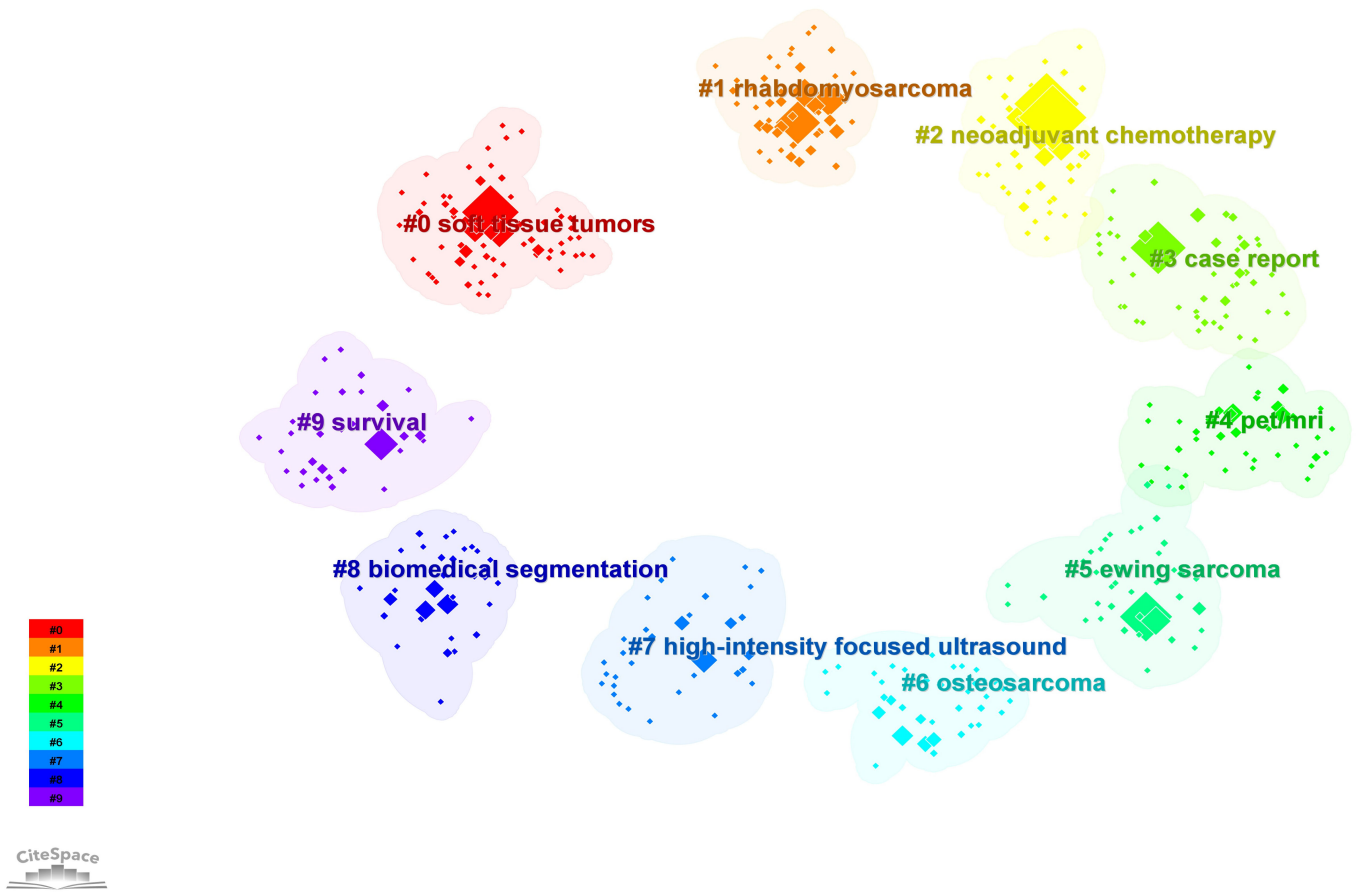
Cluster analysis of keywords of the PET/CT utility in bone and soft tissue sarcoma from 2010 to 2023.

To further explore the research hotspot and development trend of PET/CT application in the sarcoma field, we conducted cluster analysis on the keywords collected in this field. CiteSpace adopted the classical LLR algorithm to cluster the keywords closely related to each other, and finally obtained 10 clusters. Currently, the primary areas of research can be grouped into the following categories: #0 soft tissue tumors, #1 rhabdomyosarcoma, #5 Ewing sarcoma, and #6 OS are considered as indications for PET/CT in sarcoma, aiding in the diagnosis, localization, and grading of these diseases; #2 neoadjuvant chemotherapy and #7 high intensity focused ultrasound are categorized as PET/CT for monitoring the treatment response of sarcomas; #9 survival analysis using PET/CT can be utilized for prognostic prediction; #8 biomedical segmentation is classified as PET/CT combined with deep learning to enhance the diagnosis of sarcoma; #4 PET/MRI is classified as the integration of PET with other conventional imaging techniques in the management of sarcomas; and #3 case report represents a shift in the research approach employed by PET/CT in sarcomas (**Figure 6**; **Supplementary Table 5**).

The current application value of PET/CT in the field of sarcoma can be essentially summarized by these 10 clusters, which also indicate the future development direction. PET/CT is becoming increasingly essential in the management of sarcoma, progressing from initial histological grading to broader utilization in bone sarcoma and STS. It plays a crucial role in various aspects such as localization, staging, NCT guidance, efficacy assessment, and prognosis monitoring (54). Simultaneously, enhancing the precision of PET/CT will be the primary area of investigation in forthcoming studies. Can the utilization of PET and other imaging methods lead to improved imaging outcomes? ^18^F-FDG is not the only imaging agent, and tracers that can be used in integrated diagnosis and treatment are constantly being developed. These needs and issues are leading us to a shift in the direction and type of research. We discuss this in detail below.

## 4 Discussion

### 4.1 PET/CT for the initial diagnosis of sarcoma

Early and accurate diagnosis of the disease, along with assessment of its extent, are important for proper stratification of treatment and affect patient prognosis (10, 11). While histopathological examination is considered the benchmark for diagnosing sarcoma, the presence of sarcoma heterogeneity and the limited sampling materials can lead to false negatives. Moreover, there is a potential risk of contaminating surrounding tissue and causing sarcoma metastasis (55–58). Hence, it is imperative to employ more precise non-intrusive techniques to aid in the diagnosis and classification process.

The ^18^F-FDG PET/CT scan offers a non-invasive way to gather both functional and anatomical data, aiding in the detection of hidden illnesses that may have been missed by CT scans, assessing uncertain observations, and verifying metabolic activity in potential cases of metastatic diseases (13, 59, 60). Research has indicated that PET/CT has a high level of sensitivity in the initial detection of sarcoma (96.4% for soft tissue sarcoma and 96.0% for OS) (61). In a retrospective analysis by Muheremu et al., ^18^F-FDG PET/CT application based on SUVmax was found to be a valuable complementary method for histopathological detection of sarcoma diagnosis and staging (60). Since most malignant cells exhibit increased aerobic glycolysis (i.e., Warburg effect), FDG is preferentially located in these tumor cells compared to many normal tissues, showing a fanatical uptake of FDG. This important feature is the basis for determining tumor grade, quantifying therapeutic response changes, and determining prognosis. Moreover, functional changes in FDG uptake and metabolism usually precede anatomical changes, contributing to early diagnosis of the disease (62).

However, high FDG uptake does not necessarily indicate malignancy (62). Certain non-malignant bone and soft tissue abnormalities, like schwannomas, fibrodysplasia, osteomyelitis, and rheumatoid arthritis, can also cause elevated FDG absorption (9, 12, 63). Hence, the precise function of ^18^F-FDG PET/CT in the primary diagnostic assessment of OS and STS remains uncertain, and it is not advised as the initial imaging technique for STS and bone sarcomas (64).

### 4.2 PET/CT as an indicator of histologic grade

Histopathological analysis plays a crucial role in determining the grade of sarcoma, which in turn serves as a significant indicator of both sarcoma recurrence and prognosis. Additionally, it plays a pivotal role in guiding the treatment process. The use of FDG PET/CT can assist in biopsy procedures by identifying the areas of highest metabolic activity in the lesion. This helps prevent the underestimation of histological grade and overcomes the limitations of standard biopsy in lesions with high heterogeneity. As a result, it reduces the occurrence of false negative results and complications from repeated biopsy (38, 42). Furthermore, the inclusion of metabolic information obtained through FDG PET/CT, such as SUVmax, MTV, and TLG, could potentially enhance the precision of sarcoma classification and prognosis assessment. High-grade or malignant tumors have a higher TLG compared to low-grade or benign tumors (65). Furthermore, prior research has indicated that the SUVmax exhibits a positive correlation with the histological grade of bone sarcoma and STS, reaching a correlation coefficient of up to 0.94. High-grade tumors usually show an SUVmax of 10 or higher, which is higher than benign or low-grade tumors (38, 66, 67).

However, this is similar to the diagnostic mechanism mentioned earlier. The uptake of FDG also involves mitotic rate, cytosexuality, and other indicators (66). Low grade chondrosarcomas also tend to exhibit low FDG uptake rates due to low mitotic activity (6). Hence, additional extensive future studies conducted across multiple centers are required to investigate approaches for enhancing the effectiveness of PET/CT in the initial detection and classification of sarcomas. Adding randomized controlled trials of sarcoma treatment strategies on this basis will help stratify patient treatment and determine the best treatment regimen based on the stage of an individual patient.

### 4.3 PET/CT for the staging and restaging of sarcoma

Numerous studies have indicated that PET/CT surpasses CT by itself when it comes to the classification of initial tumor (stage T), related lymph node ailment (stage N), and distant spread of cancer (stage M) (68–70), thereby enhancing the classification and reclassification of sarcoma. The lung is the primary location where OS and STS commonly spread, and patients with metastases have a low 5-year survival rate of just 20%. Hence, identifying lung metastasis is crucial in the staging and restaging of sarcoma (9). Prior research has indicated that PET/CT might fail to detect tiny lung nodules because of the limited spatial precision of the CT component (thicker 5mm reconstruction segments) and the absence of breath holding during data collection. Therefore, chest specific breath-holding CT scan (BH-CT) is often the standard staging procedure for detecting distant lung metastases, and chest CT combined with FDG PET/CT is suitable for follow-up in patients with sarcomatous lung metastases (13, 71, 72). However, Flavell et al.’s research has shown that incorporating thin-core lung section (2mm) reconstruction during low-dose CT with PET examination can enhance the identification of pulmonary nodules to a similar extent as conventional BH-CT, without any extra scan time or radiation. Furthermore, this technique can be seamlessly integrated into regular clinical practice (73).

Moreover, PET/CT (sensitivity: 98%, specificity: 97%) is superior to scintigraphy and conventional imaging in detecting bone and lymph node metastases (20, 74). Nevertheless, around 80% of patients (particularly those with high-grade sarcoma or clinically suspected cases) may experience recurring illness within the initial 3 years following treatment (75). It was observed that PET/CT exhibits greater sensitivity and specificity in identifying recurrent OS or STS when compared to CT alone, with percentages of 94% and 78%, respectively (76). However, it should be noted that postoperative and post-radiotherapy states and post-traumatic inflammation may lead to false positive results that require further confirmation by CT or MRI or follow-up imaging (20, 77). Therefore, the period 6–8 weeks after treatment may be more suitable for obtaining FDG PET/CT images (13).

### 4.4 PET/CT and response to therapy

In the last decade, the treatment of malignant sarcomas has emphasized the need for preoperative NCT for high-grade tumors (78). Therefore, preoperative chemotherapy response assessment is of great significance for individualized treatment strategies (41). This information could assist in deciding whether to modify chemotherapy treatment during NCT, discontinue preoperative chemotherapy, as well as evaluate the likelihood of local recurrence following surgery (79).

In numerous cancer types, ^18^F-FDG PET/CT has become a hopeful imaging technique for predicting NCT responses at an early stage without invasive procedures (80). FDG PET/CT imaging changes may precede anatomical changes and help determine whether treatment of a particular lesion is complete, partial, or unresponsive before morphological changes are observed (81). Studies have demonstrated that a decrease of 35% in tumor ^18^F-FDG absorption following treatment, in comparison to the pre-treatment level, serves as a reliable indicator of histopathological response (PR). Furthermore, there is a notable association between higher SUVmax and postoperative histologic sarcoma progression (82, 83). Bajpai et al. utilized multivariate analysis and discovered that following three rounds of NCT, the ratio of SUVmax between post-treatment and pre-treatment was 0.48, which served as a separate indicator for histological tumor necrosis of ≥90% (84).

Moreover, ^18^F-FDG PET serves as a diagnostic tool to assess the effectiveness of cancer cell treatment through fibroblast growth factor receptors (FGFR)-targeted therapy, which is activated by FGFR and mTOR/HK2 axis. To provide a visual basis for the selection of the initial treatment plan for tumor patients and the adjustment of the treatment plan in the process of drug resistance evolution (85). Recent research has demonstrated that high intensity focused ultrasound (HIFU) can accurately and precisely deliver energy to the treatment area while minimizing harm to nearby structures (86, 87). This provides a new option for the treatment of sarcomas. The evaluation of the therapeutic effect of HIFU based on SUVmax is frequently conducted using ^18^F-FDG PET/CT. Nevertheless, there is an abundance of conflicting findings, and neoadjuvant therapy has also shown a significant decrease in FDG uptake among non-responsive individuals, necessitating careful evaluation before considering additional surgery (64).

In addition, Lin et al. (88) confirmed the clinical practicability of the imaging omics model through decision curve analysis. The delta-imaging omics histogram combined with imaging characteristics and clinical factors can be used to evaluate individual pathological reactions after preoperative chemotherapy, which is helpful to formulate appropriate chemotherapy and further treatment plans (89). In addition, Anna et al. (90) proposed a biomarker for predicting NCT PR. The researchers discovered a negative correlation between PR and the expression of HIF-1α, suggesting a suboptimal treatment response. Conversely, a positive correlation was observed between PR and the high expression of γH2AFX prior to treatment.

### 4.5 Prognostic factors of sarcoma with PET/CT

Given the typically unfavorable outlook for sarcomas, it is crucial to ascertain numerous prognostic elements to determine the optimal course of treatment and effectively plan subsequent examinations (91). Numerous studies have investigated the prognostic significance of ^18^F-FDG PET/CT in predicting survival outcomes, demonstrating its immense potential in the management and follow-up of sarcomas. A meta-analysis was performed by Li (91) and Chen et al. (92), which revealed that the prognostic value of progression-free survival and overall survival was effectively determined by SUV prior to chemotherapy, SUV post chemotherapy, SUV ratio, SUVmax, MTV, and TLG. In general, a high SUVmax indicates a poor prognosis, whereas a lower value indicates a better prognosis in most patients. Other researchers have suggested that a SUVmax (≥10.3) can independently predict both progression-free survival and overall survival. Additionally, a high SUVmax is strongly linked to a 2.9 times higher chance of sarcoma progression and a 3.2 times higher chance of mortality (9, 93). Higher TLG following treatment is linked to poorer overall survival, and individuals with extensive and irregular MTV may be considered for more intensive initial chemotherapy (24, 94).

Nevertheless, the predictive factors for localized STS are still a topic of debate, with the majority of prior investigations being retrospective and encompassing diverse populations with varying histological classifications and anatomical sites. In the absence of larger multicenter trials, the clinical value of predicting individual outcomes outside of predefined clinical situations is not feasible. There is insufficient evidence to support the clinical application of FDG PET/CT in patient outcomes and prognosis and this needs to be further explored. Therefore, large, multicenter, prospective studies in homogeneous patient populations are required to provide evidence to demonstrate and summarize specific clinical criteria for PET/CT in different sarcoma types, in different radiopharmaceuticals.

### 4.6 PET/CT combined with deep learning to improve the diagnosis of sarcoma

Current imaging techniques face difficulties in accurately assessing the extent of tumors at an early stage. Traditional medical image segmentation methods include superpixel segmentation, watershed segmentation, and active contour (28, 29, 95). Segmentation is often performed manually by different imaging experts and can be subject to errors. PET/CT enhances the precision of tumor segmentation by merging the exceptional PET tumor detection sensitivity with the anatomical details provided by CT. Nonetheless, previous automated segmentation techniques primarily concentrated on merging extracted data that is separated from PET and CT models, assuming that each model possesses supplementary information, and did not fully exploit the superior PET tumor sensitivity that can assist in segmentation (96). In recent years, with the advancement of deep learning technology, the utilization of deep learning for medical image segmentation has increasingly become a conventional method (29).

Fu et al. (96) attempted to use PET images to guide CT segmentation, introducing a deep learning-based multimodal PET/CT segmentation framework and a multimodal spatial attention module (MSAM). MSAM automatically learns to emphasize areas associated with tumors (spatial regions) and suppresses normal areas with a physiologically high uptake of PET inputs. The resulting spatial attention maps are then used to target the convolutional neural network backbone to segment areas of high tumor likelihood from CT images. Validated on PET/CT datasets for non-small cell lung cancer and STS, the method surpassed state-of-the-art lung tumor segmentation techniques by 7.6% in the Dice similarity coefficient, demonstrating superior performance.

Furthermore, owing to the high expenses associated with PET imaging, in numerous instances, only CT images can be acquired. The Siamese semi-disentanglement network was suggested by Diao et al. (29), aiming to separate medical images into anatomical and modal information. This network can achieve comparable results to full-modal segmentation even when PET images are not available. In addition, they suggested an attention network that combines PET/CT volume-based spatial compression and multi-modal feature fusion for accurately segmenting multiple tumors throughout the body. This approach effectively utilizes the fusion of contextual information from the entire body and information from multiple modalities to accurately identify tumor regions based on anatomical information of the entire body (28). In summary, especially in 2023, there are many outstanding achievements in the subdivision of tumor biology and PET/CT, which is a development trend with high potential in the future. We are confident that there will be further outstanding outcomes in the future, which will contribute to the enhanced utilization of PET/CT in clinical settings.

### 4.7 New mix: PET with other imaging techniques for sarcoma

Currently, the combination of PET and CT is considered one of the most precise techniques for tumor staging (83). However, the main limitation of PET/CT is the low soft-tissue contrast in the CT portion. By utilizing a completely integrated PET/MRI system, this limitation can be effectively addressed. PET/MRI is a hybrid imaging technology that is relatively new when compared to PET/CT (13, 83). MRI is ideal for local tumor identification and the initial assessment of relationships with neighboring organs and peripheral neurovascular structures. The comprehensive metabolic information of PET can complement MRI, especially when the tumor coexists with scar tissue, to help distinguish between surviving tumors and scars (13). Combining PET with MRI, with higher soft tissue contrast and lower radiation dose, reduces the time required for imaging alone and is a key factor for patients with tumors undergoing long-term follow-up.

MRI provides better differentiation between soft tissue and bone marrow in bone sarcoma and STS compared to CT, leading to improved evaluation of local tumor and treatment response (9). Furthermore, a prospective study showed that PET/MRI has a high potential for evaluating bone metastases by providing higher lesion significance and diagnostic confidence. However, PET/CT remains a reference modality for highly sclerotic and benign bone lesions (97). Felipe et al. also indicated that PET/MRI has higher sensitivity and specificity. Based on PET/MRI, SUV, and apparent diffusion coefficient collected using volume histograms, these are dependent biomarkers of FDG affinity sarcoma. Overall, PET/MRI has the capacity to emerge as a customary examination for the classification and assessment of numerous initial malignancies (98, 99).

In addition to MRI, DWI has also been found to more accurately forecast NCT PR when utilized alongside PET (40). Similarly, the previously mentioned HIFU is gaining prominence in sarcoma treatment. If used in combination with PET, can sarcoma be diagnosed and treated simultaneously with low radiation doses? These interesting ideas encourage us to further explore different combinations of PET and various imaging methods in the future to obtain the best imaging methods and realize the integration of diagnosis and treatment.

### 4.8 Beyond FDG: emerging PET agents

Hypoxia is an important characteristic of malignant sarcomas. In bone sarcoma and STS, hypoxia-induced gene expression patterns can be used to diagnose and predict outcomes. Additionally, STS with hypoxia has an increased likelihood of distant metastasis and reduced overall survival (9). PET and ^18^F-labeled nitroimidazoles provide specific measurements of intracellular hypoxia. Among drugs in this category, ^18^F-FMISO was the first to be extensively tested. Shorter disease-free survival and disease-specific death (9, 100) are linked to elevated uptake of ^18^F-FMISO at baseline. In addition, the same type of ^18^F-flunidazole (^18^F-HX4) is associated with tumor hypoxia (101).

Sarcoma cells utilize hypoxia and the DNA damage response as mechanisms to react to multidisciplinary treatment (90). The uptake of 3’-deoxy-3’-FLT (^18^F-FLT), which is similar to the nucleotide thymidine and serves as a marker for DNA replication, has been investigated as an indicator of tumor cell growth (94). A feverish ^18^F-FLT phenotype has been reported in various STS tissue types. ^18^F-FLT uptake is associated with tumor grade (94, 102). In addition to these aspects, ^18^F-sodium fluoride (^18^F-NaF) is also an excellent bone tracer, indicating osteoblast activity through uptake to identify reactivity changes in potentially affected bone, and is often used for imaging metastatic bone lesions (103).

Cancer-associated fibroblasts in the stroma of different types of tumors show significant expression of fibroblast activating protein (FAP), making it a potential target for diagnosis and treatment. A novel FAP inhibitor with ^68^Ga radiolabeling, ^68^Ga-FAPI-46, has shown high uptake rates in PET/CT imaging of sarcoma patients, showing great potential (94). With the popularity of the concept of integrated diagnosis and treatment, the Arg-Gly-Asp (RGD) tripeptide sequence has also been showing potential. With high specificity, it strongly attaches to integrin αvβ3 found on neovascular endothelial cells and various tumor cells, indicating its potential as a molecular agent for angiogenic imaging. Additionally, the RGD peptide has the ability to decrease the density of functional blood vessels, hinder adhesion, and trigger apoptosis in cancer cells, emerging as a novel focus for therapeutic intervention (104). In addition, Zang et al. (105) developed a double-targeted PET probe ^68^Ga-FAPI-RGD. It showed higher developer uptake and better image target/local ratio in most tumor lesions and showed more lesions, suggesting broad clinical application potential in the future.

Tumor uptake is thought to reflect increased amino acid metabolism in cancer cells, such as increased active transport and protein synthesis (106). L-[3-^18^F]-a-methyltyrosine (FMT, an amino acid analogue) accumulates in tumor cells only through the amino acid transport system (107). Watanabe et al.’s findings show that FMT PET not only shows all malignancies and a similar proportion of benign lesions detected with FDG PET, but also that FMT may be superior to FDG in the differentiation of benign and malignant musculoskeletal lesions owing to its higher sensitivity and specificity, which are important for preoperative planning (108). Choline is a vital component of cell membranes. In malignant cells, the up-regulation of choline kinase catalyzed choline phosphorylation can reflect the proliferation of cell membranes (109). In a retrospective study, researchers found that ^11^C-choline PET/CT plays an important role in staging patients with bone and soft tissue sarcomas, improving the accuracy of overall TNM staging compared to conventional imaging, and in particular providing important additional information on the staging and restaging of bone metastases in prostate cancer patients and soft tissue sarcomas (106, 110). Recently, overcoming the limitation of the short half-life of ^11^C, the gradual adoption of labeling choline compounds with ^18^F provides greater flexibility for imaging protocols and availability (111). ^18^F-choline PET/CT also has advantages in the early detection of metastatic bone disease owing to better identification of the results of bone marrow infiltration (112).

However, unfortunately, most of the current radiotracers are broad-spectrum tumor imaging agents, and there is no effective and specific radiotracer for a certain sarcoma. Moreover, the use of different therapeutic nuclide labeling tracers, such as ^177^Lu and ^225^Ac, may achieve unexpected therapeutic effects. Further investigation should prioritize the exploration of distinct radiopharmaceuticals suitable for integrated diagnosis and treatment, aiming to enhance diagnostic and therapeutic precision while minimizing harm to healthy tissues. Regarding the diagnosis and treatment of sarcoma, **Table 4** provides an overview of frequently used PET agents.

**Table 4:** Examples of PET reagents for diagnosis and treatment of sarcoma.

Nuclide	Tracer agent	Target or concentration site	Application	Main Indications	Reference
^18^F	FDG	Glucose glycolysis	Diagnosis, staging, treatment monitoring	Bone sarcoma and STS	(42)
^18^F	FMISO	Hypoxic tissue	Diagnosis, efficacy evaluation	Head and neck sarcoma	(14)
^18^F	HX4	Hypoxic tissue	Diagnosis, efficacy evaluation	Head and neck sarcoma	(101)
^18^F	FLT	DNA replication	Diagnose, assess scope, monitor for recurrence	Brain glioma	(102)
^18^F	FGFR1	FGFR	Diagnosis	Lung cancer	(113)
^18^F	NaF	Bone metastasis	Detection of bone metastasis	Metastatic tumor of bone	(103)
^18^F	FMT	Amino acid	Diagnosis, identification	Bone sarcoma and STS	(108)
^18^F^/11^C	Choline	Cell membrane	Diagnosis, staging	Prostate cancer, bone metastases, STS	(110, 112)
^18^F^/68^Ga	RGD	Integrin αvβ3	Diagnosis, treatment, prognosis	Bone sarcoma and STS	(104, 106)
^68^Ga/^177^Lu	ibandronic acid (IBA)	Bone metastasis	Detection of bone metastasis, treatment	Metastatic tumor of bone	(114)
^68^Ga	FAPI	FAP	Diagnosis, treatment, prognosis	Bone sarcoma and STS	(115)
^68^Ga	FAPI-RGD	FAP, integrin αvβ3	Diagnosis, treatment, prognosis	Bone sarcoma and STS	(105)

### 4.9 Limitations

This study offers a valuable point of reference for the academic research focal points and advancement patterns in this particular field, utilizing bibliometrics and visual analysis. However, the study has some limitations. First, only English articles and reviews from the WOS core database extended index journals were searched; thus, some high-quality literature may have been overlooked. Furthermore, owing to the varying publication dates of the articles included, the earlier findings have garnered greater focus, potentially resulting in a lack of citation data for the more recent publications. Additionally, because of the inability of CiteSpace to automatically combine keywords that have identical meanings, certain keywords with comparable meanings were merged manually, potentially resulting in variations in the size of the nodes. Finally, we did not show all the clusters but selected the most representative ones for discussion.

## 5 Conclusions

PET/CT holds significant clinical importance in the domain of sarcoma and serves a crucial function in the identification, assessment of tissue quality, determination of stage, reevaluation, tracking treatment progress, and forecasting prognosis. This aspect is also the primary area of current investigation. Furthermore, the field is developing rapidly, and the combination of deep learning breaks the traditional tumor segmentation methods of PET/CT; the application value of PET combined with other traditional imaging techniques is also worth exploring. Indeed, IBA, RGD, and other new types of radioactive tracers for sarcoma diagnosis and treatment provide new choices, promoting the development of integrated diagnosis and treatment. However, despite the satisfactory outcomes, there are limitations in the study population, high costs associated with PET/CT, and ongoing debates surrounding its use in clinical practice. Future studies should be conducted with a multicenter, prospective approach, involving a substantial number of homogenous patients, to enhance the utilization of PET/CT in the sarcoma field.

## Data availability statement

The original contributions presented in the study are included in the article/**Supplementary Material**. Further inquiries can be directed to the corresponding authors.

## Author contributions

FX: Data curation, Methodology, Writing – original draft. YZ: Investigation, Software, Writing – review & editing. XT: Formal analysis, Visualization, Writing – review & editing. JZ: Validation, Writing – review & editing. TL: Validation, Writing – review & editing. YY: Validation, Writing – review & editing. WM: Conceptualization, Supervision, Writing – review & editing. YC: Conceptualization, Funding acquisition, Project administration, Resources, Writing – review & editing.
