# Supplementary material for: A bibliometric analysis based on hotspots and frontier trends of positron emission tomography/computed tomography utility in bone and soft tissue sarcoma
Source: Front Oncol. 2024 Jun 21;14:1344643. doi: 10.3389/fonc.2024.1344643 (PMC11224451; doi:10.3389/fonc.2024.1344643)
Supplement: Supplementary file 1 [file DataSheet_1.docx]

Supplemental Data

Table 1 Top 10 countries in terms of publication volume and centrality.

| Rank | Country | NP | % of 425 | NC | AC | H-index | Centrality |
| --- | --- | --- | --- | --- | --- | --- | --- |
| 1 | USA | 139 | 32.71 | 2596 | 18.68 | 26 | 0.35 |
| 2 | PEOPLES R CHINA | 73 | 17.18 | 945 | 12.95 | 15 | 0.00 |
| 3 | GERMANY | 37 | 8.71 | 1255 | 33.92 | 19 | 0.10 |
| 4 | ITALY | 34 | 8.00 | 470 | 13.82 | 12 | 0.07 |
| 5 | JAPAN | 33 | 7.76 | 629 | 19.06 | 11 | 0.02 |
| 6 | SOUTH KOREA | 31 | 7.29 | 675 | 21.77 | 16 | 0.06 |
| 7 | ENGLAND | 21 | 4.94 | 336 | 16.00 | 11 | 0.23 |
| 8 | FRANCE | 20 | 4.71 | 600 | 30.00 | 8 | 0.10 |
| 9 | AUSTRALIA | 13 | 3.06 | 238 | 18.31 | 9 | 0.01 |
| 10 | NETHERLANDS | 13 | 3.06 | 126 | 9.69 | 7 | 0.01 |

NOTE: NP, total number of publications; NC, total number of citations; AC, average citations per item.

| Rank | Institution | NP | Centrality | Country |
| --- | --- | --- | --- | --- |
| 1 | University of Texas System | 30 | 0.09 | USA |
| 2 | UTMD Anderson Cancer Center | 28 | 0.08 | USA |
| 3 | Harvard University | 18 | 0.07 | USA |
| 4 | University of California System | 11 | 0.06 | USA |
| 5 | Memorial Sloan Kettering Cancer Center | 11 | 0.03 | USA |
| 6 | St Jude Children's Research Hospital | 11 | 0.05 | USA |
| 7 | Harvard Medical School | 10 | 0.00 | USA |
| 8 | Mayo Clinic | 10 | 0.04 | USA |
| 9 | UDICE-French Research Universities | 9 | 0.13 | France |
| 10 | David Geffen School of Medicine at UCLA | 9 | 0.00 | USA |

Table 2 Top 10 institutions in terms of publication volume and centrality.

NOTE: NP, total number of publications.

| Rank | Cited journal | Country | Frequency | IF | JCR (2023.06) |
| --- | --- | --- | --- | --- | --- |
| 1 | J NUCL MED | USA | 257 | 9.30 | Q1 |
| 2 | J CLIN ONCOL | USA | 254 | 45.30 | Q1 |
| 3 | EUR J NUCL MED MOL I | GERMANY | 245 | 9.10 | Q1 |
| 4 | AM J ROENTGENOL | USA | 204 | 5.00 | Q1 |
| 5 | CANCER-AM CANCER SOC | USA | 192 | 6.20 | Q1 |
| 6 | RADIOLOGY | USA | 178 | 19.70 | Q1 |
| 7 | CLIN CANCER RES | USA | 157 | 11.50 | Q1 |
| 8 | SKELETAL RADIOL | USA | 154 | 2.10 | Q3 |
| 9 | CLIN NUCL MED | USA | 150 | 10.60 | Q1 |
| 10 | CANCER | USA | 149 | 6.20 | Q1 |

Table 3 Relevant indicators of the top 10 cited journals.

Table 4 Top 10 cited references in terms of citation frequency and centrality.

| Frequency | References | Author and publication year | Source | References | Centrality |
| --- | --- | --- | --- | --- | --- |
| 19 | The Role of F-18-FDG-PET/CT in Pediatric Sarcoma | Harrison DJ (2017) | SEMIN NUCL MED | The Role of F-18-FDG-PET/CT in Pediatric Sarcoma | 0.16 |
| 17 | Retrospective audit of 957 consecutive F-18-FDG PET-CT scans compared to CT and MRI in 493 patients with different histological subtypes of bone and soft tissue sarcoma | Macpherson RE (2018) | CLIN SARCOMA RES | Metabolic activity measured on PET/CT correlates with clinical outcomes in patients with limb and girdle sarcomas | 0.11 |
| 16 | Prediction of tumour necrosis fractions using metabolic and volumetric F-18-FDG PET/CT indices, after one course and at the completion of neoadjuvant chemotherapy, in children and young adults with osteosarcoma | Im HJ (2012) | EUR J NUCL MED MOL I | The role of FDG PET/CT in patients treated with neoadjuvant chemotherapy for localized bone sarcomas | 0.1 |
| 16 | Prediction Model of Chemotherapy Response in Osteosarcoma by F-18-FDG PET and MRI | Cheon GJ (2009) | J NUCL MED | FDG PET/CT imaging in primary osseous and soft tissue sarcomas: a retrospective review of 212 cases | 0.1 |
| 15 | Initial Metabolic Tumor Volume Measured by F-18-FDG PET/CT Can Predict the Outcome of Osteosarcoma of the Extremities | Byun BH (2013) | J NUCL MED | Early response monitoring to neoadjuvant chemotherapy in osteosarcoma using sequential F-18-FDG PET/CT and MRI | 0.1 |
| 15 | The diagnostic and prognostic value of F-18-FDG PET/CT in the initial assessment of high-grade bone and soft tissue sarcoma. A retrospective study of 89 patients | Fuglo HM (2012) | EUR J NUCL MED MOL I | Response to chemotherapy estimates by FDG PET is an important prognostic factor in patients with Ewing sarcoma | 0.09 |
| 15 | Positron emission tomography for staging of pediatric sarcoma patients: Results of a prospective Multicenter trial | Volker T (2007) | J CLIN ONCOL | F-18-FDG PET SUVmax as an indicator of histopathologic response after neoadjuvant chemotherapy in extremity osteosarcoma | 0.09 |
| 14 | Reduction of glucose metabolic activity is more accurate than change in size at predicting histopathologic response to neoadjuvant therapy in high-grade soft-tissue sarcomas | Evilevitch V (2008) | CLIN CANCER RES | The role of Fluorine-18-Fluorodeoxyglucose positron emission tomography in staging and restaging of patients with osteosarcoma | 0.09 |
| 13 | The role of FDG PET/CT in patients treated with neoadjuvant chemotherapy for localized bone sarcomas | Palmerini E (2017) | EUR J NUCL MED MOL I | Volume-Based F-18 FDG PET/CT Imaging Markers Provide Supplemental Prognostic Information to Histologic Grading in Patients With High-Grade Bone or Soft Tissue Sarcoma | 0.09 |
| 13 | [F-18]-Fluorodeoxy-D-Glucose-Positron Emission Tomography Response Is Associated With Outcome for Extremity Osteosarcoma in Children and Young Adults | Hawkins DS (2009) | CANCER | Combination of F-18-FDG PET/CT and Diffusion-Weighted MR Imaging as a Predictor of Histologic Response to Neoadjuvant Chemotherapy: Preliminary Results in Osteosarcoma | 0.09 |

NOTE: IF: Impact Factor; JCR: Journal Citation Reports 2023.06.

Table 5 The classification of clusters.

| Number | Cluster |
| --- | --- |
| #0 | soft tissue tumors |
| #1 | rhabdomyosarcoma |
| #2 | neoadjuvant chemotherapy |
| #3 | case report |
| #4 | PET/MRI |
| #5 | ewing sarcoma |
| #6 | osteosarcoma |
| #7 | high intensity focused ultrasound |
| #8 | biomedical segmentation |
| #9 | survival |

**
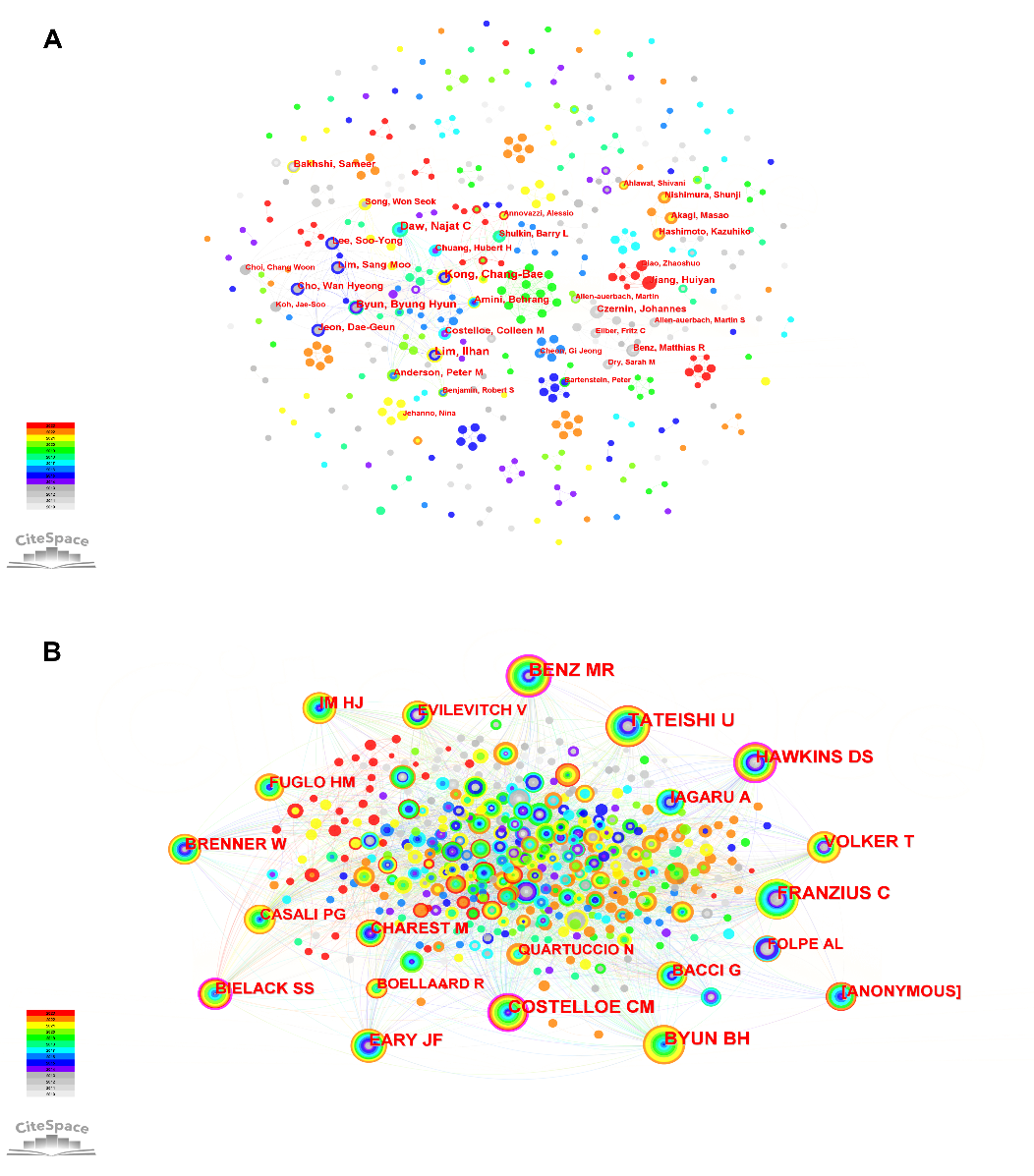
**

Figure 1 (A) Authors’ collaboration network map of the PET/CT utility in bone and soft tissue sarcoma from 2010 to 2023.(B) Co-citation network of cited authors of the PET/CT utility in bone and soft tissue sarcoma from 2010 to 2023.
